# Lipidomics and proteomics: An integrative approach for early diagnosis of dementia and Alzheimer’s disease

**DOI:** 10.3389/fgene.2023.1057068

**Published:** 2023-02-09

**Authors:** Virendra Tiwari, Shubha Shukla

**Affiliations:** ^1^ Division of Neuroscience and Ageing Biology, CSIR- Central Drug Research Institute, Lucknow, India; ^2^ Academy of Scientific and Innovative Research (AcSIR), Ghaziabad, India

**Keywords:** Alzheimer’s disease, lipidomics, proteomics, diagnosis, high throughput techniques

## Abstract

Alzheimer’s disease (AD) is the most common neurodegenerative disorder and considered to be responsible for majority of worldwide prevalent dementia cases. The number of patients suffering from dementia are estimated to increase up to 115.4 million cases worldwide in 2050. Hence, AD is contemplated to be one of the major healthcare challenge in current era. This disorder is characterized by impairment in various signaling molecules at cellular and nuclear level including aggregation of Aβ protein, tau hyper phosphorylation altered lipid metabolism, metabolites dysregulation, protein intensity alteration *etc.* Being heterogeneous and multifactorial in nature, the disease do not has any cure or any confirmed diagnosis before the onset of clinical manifestations. Hence, there is a requisite for early diagnosis of AD in order to downturn the progression/risk of the disorder and utilization of newer technologies developed in this field are aimed to provide an extraordinary assistance towards the same. The lipidomics and proteomics constitute large scale study of cellular lipids and proteomes in biological matrices at normal stage or any stage of a disease. The study involves high throughput quantification and detection techniques such as mass spectrometry, liquid chromatography, nuclear mass resonance spectroscopy, fluorescence spectroscopy *etc.* The early detection of altered levels of lipids and proteins in blood or any other biological matrices could aid in preventing the progression of AD and dementia. Therefore, the present review is designed to focus on the recent techniques and early diagnostic criteria for AD, revealing the role of lipids and proteins in this disease and their assessment through different techniques.

## Introduction

The term “neurodegenerative diseases” refers to a class of conditions in which the central and peripheral (CNS and PNS) nervous systems’ structural and functional activity gradually deteriorate (Association 2018). Although numerous laboratories throughout the world have made significant efforts, there is little that can be done for a patient who develops one of these fatal and disabling illnesses, either academically or industrially. The prevalence of dementia patients is rising, which has a severe impact on families, communities, and healthcare systems all across the world. Dementia is a term referring to brain syndromes altering memory, behaviour and emotions. The dementia subtypes encompasses AD, vascular dementia, Lewy body and fronto-temporal dementia. They exhibit the most severe form of cognitive impairment, are the leading cause of older disability, and currently impact up to 50 million people globally (Wallace and Brayne 2022). If the age-specific prevalence of dementia remains unchanged, this number is projected to rise to more than 130 million people by 2050. (Prince et al., 2019).

There has been a shift in emphasis to identify people far earlier in the disease process due to the realisation that urgent action must be made to lessen the burden of AD, a disorder with rising expenses and relatively few treatment choices. There is an urgent need to increase diagnosis rates so that those most at risk can be identified early and actions taken to slow or stop further progression. Not all people with mild cognitive impairment will develop dementia, and even though there is currently no treatment to prevent or cure the disease (Rasmussen and Langerman 2019). The ability of patients to take preventive measures before permanent brain damage occurs is dependent on an early and accurate diagnosis of AD, since they are aware of the condition’s severity and risks for progression. Despite the large number of research that have recently employed machine learning methods for AD computer-aided diagnosis, most of these studies have revealed a bottleneck in the diagnosis performance, primarily because of the inherent constraints of the chosen learning models (Liu et al., 2014).

AD incidence and, consequently, its socioeconomic effect is rising as life expectancies rise in developed nations. Since the pathomechanisms underlying AD have become better understood in recent years, targeted therapy approaches aimed at delaying or even preventing neuronal death in AD have been developed. Since non-AD dementias would not benefit from an AD-specific treatment, this also necessitates that: 1) AD can be diagnosed with high accuracy, 2) AD can be identified in its earliest stages, when any treatment would be most successful, and 3) It is possible to consistently and effectively measure a treatment’s effectiveness. Cerebrospinal fluid (CSF), blood, and neuroimaging biomarkers might be used in conjunction to provide crucial supplementary data and help in the earlier and more accurate diagnosis of AD, despite the fact that there is currently no ideal biomarker that would satisfy all these requirements (Mueller et al., 2005).

The correlations of proteins with AD and AD endophenotypes have been disclosed and replicated in numerous untargeted and targeted blood biomarker studies over the past 10 years. Brain atrophy, cognitive decline rate (CDR), and amyloid load are some of these endophenotypes. Several proteins, particularly those involved in inflammation and the complement pathway as well as complement protein C6, C-C motif chemokine, have been repeatedly associated to AD or AD endophenotypes, even though the majority of protein biomarkers have not survived further validations.

The involvement of lipids in AD have been highlighted by a number of untargeted and targeted blood metabolomics investigation. Numerous lipids still remains to be identified and quantified by lipidomics. It is acknowledged as a subset of metabolomics, exhibiting functional networks of subsequent alterations in the genome, transcriptome, and proteome, and reducing the phenotype-genotype gap, due to their close links to cellular function. When compared to controls, it has repeatedly been discovered that mild cognitive impairment (MCI) and AD have altered phospholipid cholines (PCs), cholesteryl esters (ChEs), and triglycerides (TGs). Only a small percentage of biomarker studies have used systems biology methods, and the majority have been limited to one modality (proteomics or metabolomics).

The development of large-scale sequencing methods in genomes and proteomics has sped up AD research discoveries (Metzker 2010; Aebersold and Mann 2016). Early genetic investigations identified ApoE4 as the primary AD risk gene in 1993, along with APP, PSEN1, and PSEN2. More than 160 potential risk loci connected to amyloid, tau, endocytosis, and immunology were discovered by recent high-throughput genetic/genomic study, including TREM2 and UNC5C (Jansen et al., 2019; Kunkle et al., 2019). The Accelerating Medicines Partnership (AMP)-AD programme was launched by the National Institutes on Aging in 2014, with the goal of utilising multidisciplinary methodologies from academia and industry to find new treatment targets and biomarkers. The multi-omics approach provides a crucial, organised tool for comprehending the complexities of AD (Bellenguez et al., 2020; Bai et al., 2021).

For the diagnosis of AD, clinical proteomics enables the detection of different proteins in fluids such as the urine, plasma, and cerebrospinal fluid. Plasma testing for several lipid biomarkers has increased interest in lipidomics and may work better with clinical proteomics to diagnose early brain ageing, which is linked to other chronic disorders. Proteomics and lipidomics together may reduce biological variability across investigations and produce repeatable findings that identify an AD-vulnerable population (Martins 2016). The use of genetics in the diagnosis of chronic AD-related disease may boost sensitivity and help prevent mistakes when comparing CSF fluid and plasma for disease biomarker discovery by proteomics and lipidomics in body fluids, cells, and tissues. It may now be necessary to incorporate lipidomics and genetics to provide interpretation of the proteome results from multiple laboratories around the world in order to diagnose AD using different plasma biomarkers and clinical proteomics.

### Alzheimer’s disease (AD)

Neurodegeneration due to AD underlies the majority of dementia, affecting most 50%–60% of people. Ageing stands as the substantial risk factor for this disease. AD is considered to be an intensifying a neurodegenerative condition characterised by disturbance of neural function and progressive decline in cognition, function, and behaviour (Magalingam et al., 2018). Since German psychiatrist Alois Alzheimer first identified it in 1901, AD has emerged as a major health concern, particularly for people 65 years and older (Hippius and Neundörfer 2022). The patient, a woman identified in his study as Auguste D., represented several fundamental characteristics of the AD that are still prevalent in most patients today: memory loss that is progressing rapidly; disturbed cognitive function; alteration in behaviour that includes paranoia, deprived social appropriateness and language function and delusions (Gupta et al., 2010).

During the starting phases of this disorder, the patient’s alertness and motoric/sensory functions are not significantly affected. However, the cognitive and motor functions such as gait and coordination functioning declines further that resembles the motor functioning disorder known as parkinsonism (Nestor et al., 2004). The course of AD can be described in several stages, with a progressive pattern of cognitive and impairment of functioning (Karlsson et al., 1989). Early symptoms show short term memory loss and difficulty in acquiring information and recently learnt fact. In brief, the symptomatic progression of AD incorporates three phases i.e., preclinical phase, patients have a normal cognitive behaviour although brain pathology is being somewhat altered; The second is mild cognitive impairment (MCI), which is distinguished by the existence of cognitive deficit symptoms and indicators resulting from fully established brain pathology without impairing everyday routines. The last phase known as dementia which is almost progressively impaired cognitive functioning with affecting the daily life activities (Ohm et al., 1995; Sperling et al., 2011).

### Early diagnosis of Alzheimer’s disease

The conventional clinical diagnosis of AD involves cerebrospinal fluid (CSF) assays, computed tomography (CT) or magnetic resonance imaging (MRI) which is recommended for the routine evaluation. Neuroimaging helps to analyse structural data (De Leon et al., 2004; Chu 2012). Nonetheless, the structural alterations could only be detected visually at a very late phase of the disease. Serial volumetric imaging and voxel compression subtraction are two more modern structural imaging techniques that emphasise a quantitative approach that can help in the detection of tiny changes that are difficult to detect in regular pictures taken at a single time point (Laske et al., 2015).

The same is true for functional imaging techniques such as Positron Emission Tomography (PET), Single Photon Emission CT (SPECT), and functional magnetic resonance imaging (MRI), all of which show physiologic changes in the brain (Valotassiou et al., 2018). These techniques have an equal or better potential compared to structural imaging modalities like CT and conventional MRI because they may be able to identify more subtle pathologic alterations sooner during the course of illness (Gupta et al., 2010).

### Role of lipid and protein in Alzheimer’s disease and their assessment

Notwithstanding many traits, alteration in lipid composition is also an important facet for ageing (Fabelo et al., 2014). Neuronal cell membranes are basically build-up of lipids, appraising them as the basic structural component. The lipids present in brain are comprised of phospholipids (50%), glycolipids (<40%), cholesterol (10%), and esters followed by triglyceride traces. Further, the 25%–30% of total fatty acids (FAs) is composed of long-chain polyunsaturated fatty acids (LC-PUFAs) in the human brain that includes arachidonic acid (AA) and docosahexaenoic acid (DHA) (SoOderberg et al., 1992). Moreover, the cerebral lipids reports for 50% of total dry weight and brain lipid peroxidation and changes to fatty acids at the level of lipid rafts have been observed in the early stages of AD. Cerebral lipid peroxidation was found to be an early event in AD (Lemkul and Bevan 2011). There are more lipoid granules (also known as adipose inclusions) in the glia of AD patients’ brains, which may indicate abnormal lipid metabolism. Apolipoprotein E (ApoE clusterin (also known as apolipoprotein J), SORL1 (sortilin-related receptor 1), and ABCA7 ((ATPbinding cassette, sub-family A, member 7) are a few of the genes involved in lipid homeostasis that have been linked to AD in genome-wide association studies (GWAS) (Kao et al., 2020).

The classical pathological hallmark of AD includes depositions of several which are amyloid beta (Aβ) protein as extracellular senile plaques and intracellular accumulation of neurofibrillary tangles (NFTs) and tau proteins. The major enzymes and proteins that contribute to the course of AD include amyloid-β, β-secretase, tau protein, monoamine oxidases, and methionine sulfoxide.

### Assessment of proteins involved in Alzheimer’s disease proteomics

Proteomics can be defined as “the large-scale characterization of the entire protein complements of a cell line, tissue, or organism”. Today, two definitions of proteomics are confronted. The first is the more traditional definition, which limits investigations involving only proteins to a large-scale examination of gene products. The second, broader definition integrates genetically based investigations with protein studies, including mRNA, genomic, and yeast two-hybrid analyses (Tyers and Mann 2003; Swomley et al., 2014). However, proteomics’ objective is still to investigate all of a cell’s proteins rather than just each one separately in order to gain a more comprehensive and integrated understanding of biology (Graves and Haystead 2002). To ascertain alterations in the amount of protein expression of the brain’s proteins in connection to the mutation that makes the rat a useful model for AD, several proteomic techniques have been used. Additional research on crucial proteins in AD has been carried out by isolating the protein of interest from the AD brain using preparative gel electrophoresis and gel electroelution, which were then analysed using mass spectrometry. Following two important studies that are Focused upon: apoE4 and β-tubulin. Hesse and others described apoE4 isolated from AD cerebrospinal fluid (CSF) and verified using MS analysis the known change of Cys to Arg at position 112 of apoE4, which reflects the increased frequency of the apoE4 allele in the AD population (Hesse et al., 2001). To elucidate the state of β-tubulin isolated from AD brain, a similar investigation was conducted (Vijayan et al., 2001; Barbier et al., 2019). β -Tubulin was discovered by mass spectrometry analysis to be an abnormally hyperphosphorylated protein in AD brain, supporting the theory that it participates in the breakdown of the microtubules (MT). In AD, brain tubulin loses its capacity to assemble MTs; however, when dephosphorylation takes place, this capacity is restored (Wandosell et al., 1986; Wang et al., 1994). Even a little amount of changed tubulin may be crucial for the disintegration of MT in the AD patient’s brain.

Understanding the proteins present in the hippocampus may help explain the known pathological alterations. In a study by Sultana and others they compared the protein levels in AD and control hippocampus using two-dimensional gel electrophoresis and mass spectrometry techniques. They discovered 18 proteins that are involved in controlling various cellular processes and have changed protein levels. Phosphoglycerate mutase 1 (PGM1), dihydropyrimidinase-like protein 2 (DRP-2), beta-III tubulin (0.34-fold compared to control, *p* < 0.01), and aldolase A (0.87-fold compared to control, *p* < 0.0002) were found to have significantly decreased protein levels, whereas the protein levels were found to be significantly increased for the other proteins. They discovered two locations on proteins that were glyceraldehyde 3-phosphate dehydrogenase (GAPDH). One of the locations had a protein level that was 1.28 times higher than the control (*p* < 0.01), while the other had a protein level that was 1.26 times higher than the control (*p* < 0.04). The amounts of important proteins in the AD brain have therefore been determined with the help of proteomics (Sultana et al., 2007).

One of the most thorough analyses of the serum proteome is possible with the TMT-LC/LC-MS/MS (11-plex tandem-mass-tag) platform, which can analyse 4,826 protein components (4,368 genes), covering at least 6 orders of magnitude in dynamic range. In the AD and control groups, Dey and others defined intra- and inter-group variability. This statistical study uncovered proteins that were expressed differently in AD (26 decreased and 4 increased). Notably, the known pathways of mitochondria, fatty acid beta oxidation, and AGE/RAGE were found to be concentrated in these changed proteins. In order to corroborate the decline of PCK2 and AK2 in the AD samples, they performed a multiplexed targeted LC–MS3 method (TOMAHAQ) technique (Dey et al., 2019). Moreover, Dey et al., 2022 recently highlighted that with the development of multiplexed tandem mass tag labelling combined with two-dimensional liquid chromatography and tandem mass spectrometry (TMT-LC/LC-MS/MS), mass spectrometry (MS) has emerged as a popular platform for comprehensive proteome characterization. Recently, using the 16-plex TMTpro method, their group have developed a reliable procedure for directly profiling the proteome of undepleted cerebrospinal fluid (CSF). To do this, they optimised various experimental parameters during the steps of sample preparation, TMT labelling, LC/LC fractionation, tandem mass spectrometry, and computational data processing. The substantial LC fractionation improves CSF proteome coverage while also reducing TMT quantification ratio distortion. The essential quality assurance procedures and adjustments unique to the TMT16 study were outlined. A single experiment could quantify more than 3,000 proteins from 16 distinct CSF samples. A potent tool for profiling a range of complicated biofluid samples, such as CSF, serum/plasma, and other clinical specimens, was provided by this multiplexed approach (Dey et al., 2022).

**FIGURE 1:**
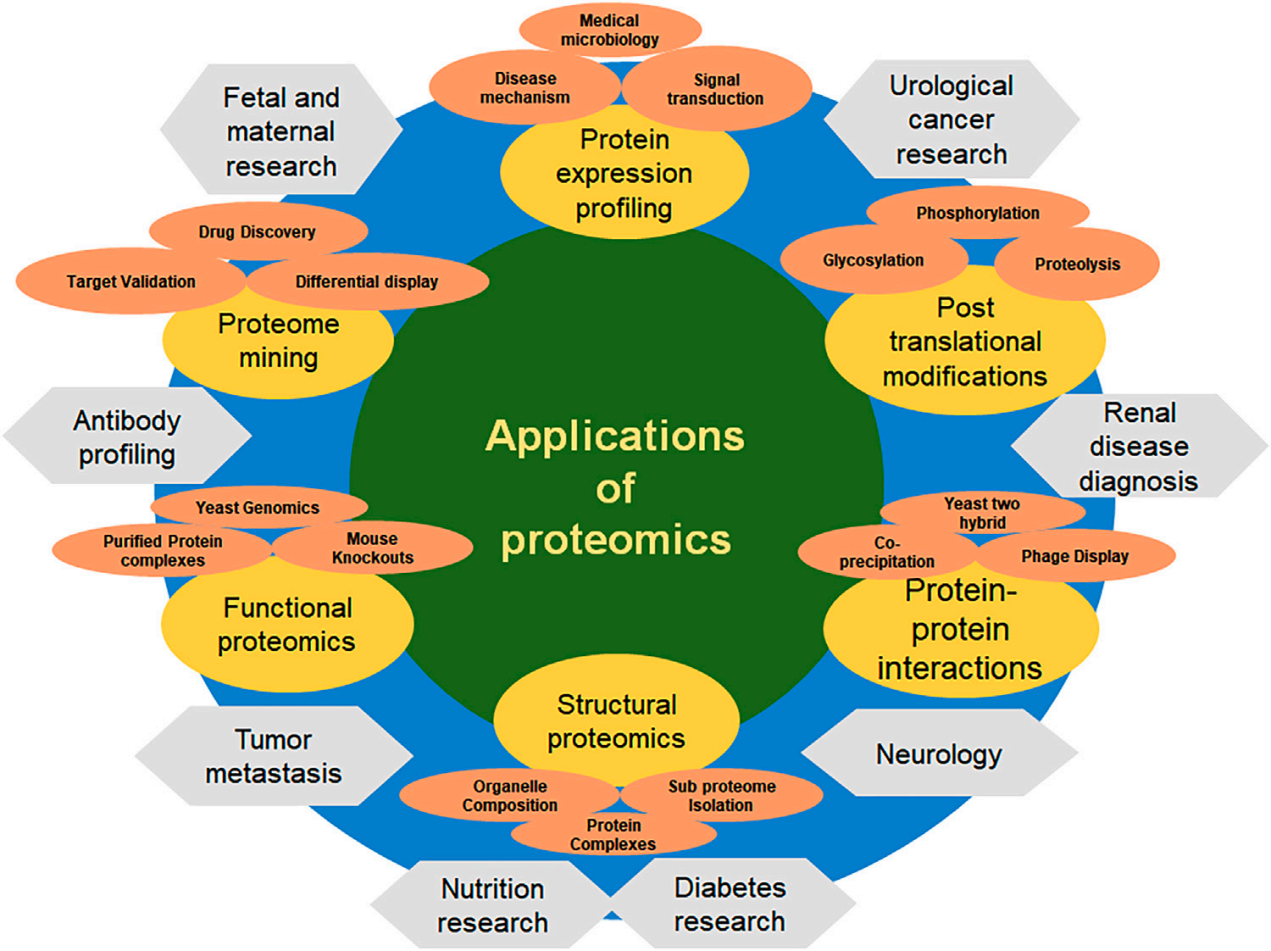
Applications of proteomics.

Proteomics is applied to investigate the following analysis (Figure 1):

*Protein localization:* As cellular localization determines which molecular interaction partners and targets are available, the location of a protein during its expression and/or accumulation is just as important to its function as the timing of expression.

*Post-translational modifications:* Post-translational modifications can alter the stability, interactions, activation, localisation, and signal transduction of proteins, among other protein properties, adding a considerable level of biological complexity.

*Functional Proteomics:* The focus of this branch of proteomics is on determining the biological roles of particular proteins, protein subclasses (such as kinases), or entire protein interaction networks.

*Structural proteomics:* Insights into protein function, the “druggability” of protein targets for drug discovery, and drug design can all be gained through structural investigations.

*Protein-Protein interactions:* examines the interactions between different proteins, their types, locations, and times of interaction.

### Protein involved in Alzheimer’s disease

**FIGURE 2:**
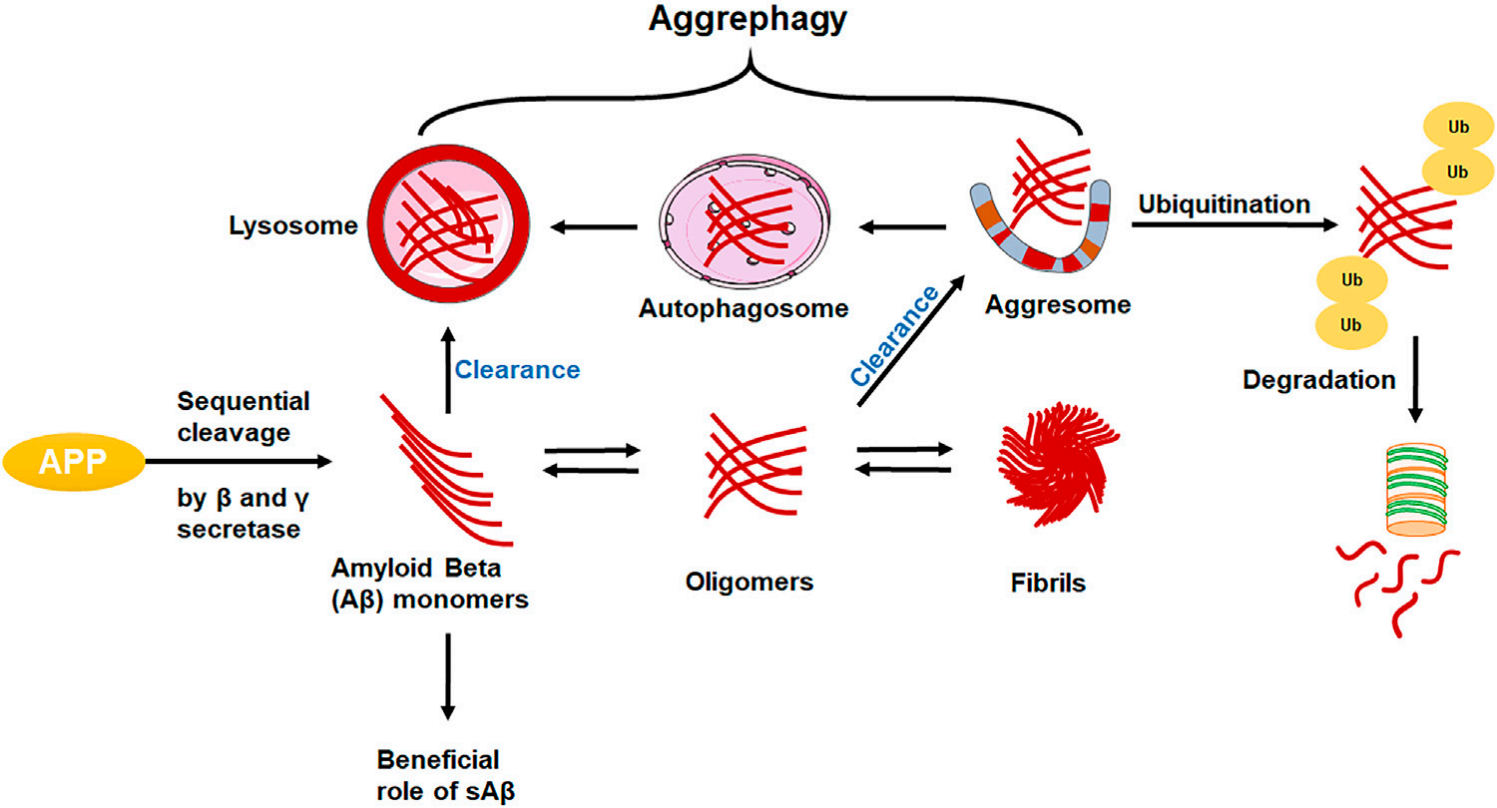
Assessment of protein in AD brain.

The classical pathological hallmark of AD includes depositions of several proteins which are, amyloid beta (Aβ) protein as extracellular senile plaques and intracellular accumulation of neurofibrillary tangles (NFTs) and tau protein (Figure 2) (Kolarova et al., 2012). Aβ, secretases, tau protein, monoamine oxidases, and methionine sulfoxide reductase are some of the distinctive enzymes and proteins that are implicated in the course of AD. (Irizarry 2004; Roychaudhuri et al., 2009).

• APP (Amyloid Precursor Protein)
• Aβ (Amyloid Beta)
• β-secretase
• α-secretase
• γ-secretase
• Tau NFT (Neurofibrillary tangles)
• ApoE (ApoEnzyme)

*APP:* Here, the APP (Chr 21q21) gene encodes a type 1 transmembrane protein that is widely expressed and has three main splice variants: APP695, APP751, and APP770. The predominant isoform, APP695, is expressed in neurons, while the splice variation, APP751, is primarily found in astrocytes. Cell-surface APP is absorbed, enabling endocytic pathways to digest it, and various fragments are released into the extracellular space (Zhou et al., 2011). The majority of APP is processed in a non-amyloidogenic manner by successively being cut by sAPP and C-terminal secretases in the A domain (CTFs). Instead, APP is sequentially cleaved by the β- and γ-secretases to produce the neurotoxic A peptides, as well as sAPP and CTF. (Yoshikai et al., 1990). The amino acid composition of the amyloid precursor protein is altered by the most prevalent APP gene variation. In this variation, the amino acid valine is swapped out for the amino acid isoleucine at position 717 in the protein (written as Val717Ile or V717I). The amyloid peptide can be produced in greater amounts or in a slightly longer and stickier form as a result of variations in the APP gene (Müller and Zheng 2012; Walter and van Echten-Deckert 2013).

*Amyloid beta:* The level of Aβ42 in cerebrospinal fluid (CSF) starts to decline in the early stages of AD. (Shaw et al., 2009), while the concentration of Aβ42 in the brain is rising (Steinerman et al., 2008), suggesting a decrease in Aβ transport from the brain, with a recent metabolic investigation of brain Aβ clearance in humans providing substantial support for this hypothesis (Mawuenyega et al., 2010). As an alternative, a change in the brain’s ratio of Aβ-42 to Aβ-40 or any alteration in the creation of CSF or the molecules that buffer Aβ in CSF, including ApoE, may cause greater Aβ-aggregation and reduced CSF clearance.

*Tau:* Tau is the microtubule-associated protein (MAP), forms insoluble filaments that accumulate as neurofibrillary tangles (NFTs) in AD and related tauopathies. Under physiological circumstances, tau controls the formation and repair of microtubules’ structural stability (Kolarova et al., 2012). However, tau is abnormally hyperphosphorylated in diseased brains, leading to the disintegration of microtubules and the formation of paired helical filaments from free tau molecules (Chong et al., 2018). A substantial body of research indicates that tau hyperphosphorylation is caused by cellular signalling disruption, primarily due to an imbalance in the activity of several protein kinases and phosphatases. It indicates that β-amyloid peptide (Aβ) is crucial in causing this imbalance in AD (Iqbal et al., 2010).

*ApoE enzymes:* Brain lipoproteins with ApoE (ApoEnzyme) are responsible for phospholipid and cholesterol transport. There are three primary isoforms of ApoE, ApoE2, ApoE3, and ApoE4, which are mostly expressed in astrocytes and microglia and are the biggest genetic risk factors for AD (Schaduangrat et al., 2019). Additionally, boosting the expression of these receptors may be a therapeutic strategy for the treatment of AD because levels of ApoE LDL receptors directly correlate with the clearance of Aβ (Penke et al., 2019).

Because of recent developments in proteomics methods, the CSF proteome has now been estimated, with measurements containing up to 3,000 distinct proteins at once in humans. CSF protein levels fluctuate, indicating continuous biological processes, which may be useful for future research on the pathophysiology of AD. A subsequent CSF proteomics meta-analysis which included 48 different neurological disorders in addition to 18 studies on AD reported that 48 proteins were specifically dysregulated in AD compared to other neurodegenerative illnesses, and that 309 proteins exhibited altered levels in AD compared to controls. The enrichment of these AD-associated proteins for pathways related to steroid esterification, protein activation cascade (i.e., a pathway composed of immune and haemostasis-related genes), and negative regulation of response to external stimuli supports the hypothesis that biological complexity may be reflected in the CSF proteome. Since about 10% of people with AD-type dementia do not have aggregated amyloid, although they might not accurately reflect the presence of AD pathology, studies that defined AD based on clinical dementia diagnosis were non-etheless included. (Ossenkoppele et al., 2015; Bastos et al., 2017). In the quantification and identification of biological macromolecules like proteins, peptides, and nucleic acids using mass spectrometry (MS), sample multiplexing is made easier by the use of tandem mass tags (TMTs), which are chemical labels. Tandem mass tag (TMT) proteomics is a potent method for biomarker identification since it allows for the simultaneous quantification of hundreds of proteins in large cohorts. TMT proteomics in CSF, however, is accompanied by analytical difficulties in sample preparation and data processing (Weiner et al., 2022). Many researchers have employed the TMT based technique using TMT labeling with SPS-MS3, 27-plex TMT approach to profile the intricate human brain proteome of AD after combining it with two-dimensional liquid chromatography (LC/LC) for comprehensive peptide fractionation and high-resolution tandem mass spectrometry (MS/MS) for peptide quantification (Li et al., 2020) and to obtain a core group of highly abundant microglial proteins and a highly pure microglial proteome in adult mouse brain (Rayaprolu et al., 2020).

In a recent investigation by Wang et al., 1994, Wang and others have quantified a total of 13,833, 5,941, and 4,826 proteins from human cortex, CSF and serum, respectively. They analyzed 17,541 proteins (13,216 genes) in 365 AD, mild cognitive impairment (MCI) and control cases. The ultra-deep CSF profiling of 20 cases performed by them reported statistical differences in SMOC1 and TGFB2 proteins. Integration of 4 cortical and 4 CSF cohort proteomes revealed 6 CSF biomarkers that were consistently found in at least 2 different datasets (SMOC1, C1QTNF5, OLFML3, SLIT2, SPON1, and GPNMB). Further, they have also analysed CSF in the 5xFAD mouse model in order to confirm the amyloidosis-induced alterations and discovered comparable mitochondrial reductions (SOD2, PRDX3, ALDH6A1, ETFB, HADHA, and CYB5R3) in both human and animal samples. Finally, by merging all proteome datasets, they have identified the most promising AD hallmark proteins, such as SMOC1, TAU, GFAP, SUCLG2, PRDX3, and NTN1 (Li et al., 2020). Furthermore, in another research, the utilization of multilayer omics approach discerning various protein networks (such as MDK, NTN1, SMOC1, SLIT2, and HTRA1) during progression of AD by profiling 14,513 proteins and 34,173 phosphosites in the human brain with mass spectrometry (Bai et al., 2020) (Table 1).

**TABLE 1:** Various Techniques used in Proteomics along with their applications.

Technology	Applications	Pros	Cons	References
2DE (Two-dimensional gel electrophoresis)	Protein separation (soluble proteins of mouse tissues and the proteins of mouse serum). Quantitative expression profiling.	Relative quantitative PTM information.	Poor separation of acidic, basis, hydrophobic and low abundant proteins.	Klose 1975; O'Farrell 1975
DIGE (Fluorescence 2D difference gel electrophoresis)	Protein separation. Quantitative expression profiling identify a direct serum proteins link between Alzheimer Disease and Chronic Periodontitis Identification and validation of novel CSF biomarkers for early stages of Alzheimer’s disease. Plasma protein profiling for potential biomarkers in the early diagnosis of Alzheimer’s disease. Differential diagnosis of Creutzfeldt–Jakob disease using cerebrospinal fluid and Parkinson disease subjects. Proteomic profiling of exosomal proteins for blood-based biomarkers in Parkinson’s disease.	Relative quantitative PTM information. High sensitivity Reduction of integral variability.	Proteins without lysine cannot be labelled. Requires special equipment for visualization and fluorophores are very expensive.	Van den Bergh and Arckens 2005; Hu et al. (2007), Brechlin et al. (2008), Maarouf et al. (2012), Kitamura et al. (2017), Kitamura et al. (2018), Rong et al. (2020)
ICAT (Isotope-coated affinity tag)	Chemical isotope labelling for quantitative proteomics. Quantitative proteomic analysis of Lewy body formation. Subcellular proteomic. Quantitative proteomics of cerebrospinal fluid from patients with Alzheimer disease. Urine proteomics analysis.	Sensitive and reproducible. Detect peptides with low expression levels.	Proteins without cysteine residues and acidic proteins are not detected.	Jin et al. (2005), Zhang et al. (2005), Shiio and Aebersold 2006; Li and Smit 2008; Sobhani 2010
SILAC (Stable Isotope Labelling with Amino Acids in Cell Culture)	Direct isotope labelling of cells. Differential expression pattern to investigate the impact of amyloid precursor protein expression in neuronal-like B 103 cells. Proteome and Phosphoproteome changes in Human LRRK2 (R1441C) *Drosophila* Model of Parkinsons Disease. Identification of proteins involved in microglial endocytosis of α-synuclein.	Degree of labelling is very high. Quantitation is straightforward.	SILAC labelling of tissue samples is not possible.	(Chaput et al., 2012; Hark and Savas 2021) (Liu et al., 2007; Islam et al., 2014)
iTRAQ (Isobaric tags for relative and absolute quantification)	Isobaric tagging of peptides. Quantitative proteomic analysis of serum proteins in patients with Parkinson’s disease. Quantitative characterization of glycoproteins in neurodegenerative disorders.	Multiplex several samples. Relative quantification High-throughput.	Increases sample complexity. Require fractionation of peptides before MS.	(Zhang et al., 2012; Shi et al., 2013; Ji et al., 2019; Zou et al., 2019)
MUDPIT (Multidimensional protein identification technology). Protein array	Identification of protein-protein interactions. Detection of biomarkers with a multiplex quantitative proteomic platform in cerebrospinal fluid of patients with neurodegenerative disorders. Deconvolve complex sets of proteins. Quantitate specific proteins used in diagnostics (biomarkers or antibody detection) and discovery research. Fluid biomarkers in Alzheimer disease cerebrospinal fluid potential biomarkers.	High separation. Large protein complexes identification. High-throughput. Highly sensitive. Low sample consumption.	Not quantitative. Difficulty in analyzing the huge data set. Difficult to identify isoforms. Limited protein production. Poor expression methods. Availability of the antibodies. Accessing very large numbers of affinity reagents.	(Abdi et al., 2006; Tamura et al., 2009) (Carrette et al., 2003; Ho et al., 2005; Blennow et al., 2012)
Mass spectrometry	Primary tool for protein identification and characterization. Detection of purified amyloid beta protein robust measurement of neurodegenerative disease biomarkers in biological fluids direct analysis of peptides and proteins on brain tissue sections metabolic profiling of Parkinson’s disease.	High sensitivity and specificity. High-throughput. Qualitative and quantitative.	No individual method to identify all proteins. Not sensitive enough to identify minor or weak spots. MALDI and ESI do not favor identification of hydrophobic peptides and basic peptides.	(Mori et al., 1992; Pierson et al., 2004; Cilento et al., 2019; Korecka and Shaw 2021; Shao et al., 2021)
Bioinformatics	Analysis of qualitative and quantitative proteomic data. Discovering new genes in the pathways of common sporadic neurodegenerative diseases analysis of NGS-derived coding and non-coding RNAs in neurodegenerative diseases.	Functional analysis, data mining, and knowledge discovery from mass spectrometric data.	No integrated pipeline for processing and analysis of complex data. Search engines do not yield identical results.	(Guffanti et al., 2014; Kim et al., 2016)

### Limitations of proteomics

Contrary to the study of DNA, studying proteins has a number of special difficulties. For instance, proteins do not have a PCR equivalent, making it difficult to analyse low-abundance proteins. Additionally, to get significant results from investigations of protein interactions, native protein conformations must be preserved. Could proteins be investigated quickly, sensitively, and consistently on a wide scale? Recognition of proteomics’ limits has started to nudge the science in new directions over the past few years.

The majority of proteomics relies on low-throughput techniques like protein purification or PAGE. Even performing MS can take a lot of time during data collection and analysis. A MALDI-TOF mass spectrometer can swiftly and quickly determine hundreds of proteins, however the quality of the data is compromised and many proteins cannot be identified. By using MS/MS, much higher quality data can be produced for protein identification; however, data interpretation necessitates a considerable amount of time with this method.

The analysis of low abundance proteins presents a significant problem for proteomics. Low-copy proteins include several significant groups of proteins (that may be significant therapeutic targets), including transcription factors, protein kinases, and regulatory proteins. Without some sort of purification, these low-copy proteins will not be visible in the examination of raw cell lysates. Therefore, new techniques for sub proteome isolation must be developed (Graves and Haystead 2002).

### Lipidomics in Alzheimer’s disease

Lipids are made up of a wide range of chemically unique molecules that have different backbone structures and are made up of combinations of long chain fatty acids that are soluble in organic solvents but not in water. Currently, lipids are divided into eight categories that each comprise several types and subclasses of molecules: fatty acyls, glycerolipids, glycerophospholipids, sphingolipids (also linked to mitochondrial dysfunction in type 2 diabetes and insulin resistance) (Roszczyc-Owsiejczuk and Zabielski 2021), sterol lipids, prenol lipids, saccharolipids, and polyketides. Long chain hydrocarbons, alcohols, aldehydes, fatty acids, their derivatives (glycerides, wax esters, phospholipids, glycolipids, sulfolipids, and fatty acid esters), fat-soluble vitamins (A, D, E, and K), carotenoids, and sterols are some examples of lipids that are derived from living creatures. Based on the composition of their hydrophobic and hydrophilic groups, lipids are presently categorised into these eight groups, which are typically subdivided into neutral or polar lipids. Lipids make up around half of the brain’s dry weight. They are crucial for a variety of brain processes, including signal transduction, membrane structure, and biological messenger activities. Therefore, changes in brain lipid concentrations may represent physio-pathologic processes. (Fonteh et al., 2006). Lipidomics has been increasingly being employed to investigate lipid dysfunction in conjunction with clinical treatment and to offer important information for the pathophysiology of various disorders (Zhang et al., 2018). Systems-level investigation and characterization of lipids and their interacting components is known as lipidomics. Because of the complexity of lipids and the limitations of available techniques for analysis, the amount of information in the domains of genomics and proteomics is greater than that in the field of lipidomics (Adibhatla et al., 2006).

With the advent of non-targeted blood metabolomic investigations using direct infusion mass spectrometry (MS) or liquid chromatography-mass spectrometry (LC-MS) to study AD, the importance of lipid molecules such as sphingolipids, bile acids, desmosterol and phosphatidylcholines (PTCs) (Proitsi et al., 2015). Through cell biology and genetic investigations, lipid metabolism has been strongly linked to the aetiology of AD. In biomedical and pharmacological research, relationships with blood metabolites have been shown to be useful as functional intermediate phenotypes. Detection of lipid biomarkers for the early stages of AD could be greatly aided by research into the dynamic changes in lipidome in early-stage AD mice. By combining ultra-high performance liquid chromatography and quadrupole-time-of-flight mass spectrometry, an untargeted lipidomic method could be established for the goal of characterising lipids (1,200 Da) disturbance occurring in plasma and brain in early-stage AD mice (2, 3 and 7 months) (Proitsi et al., 2017). In a research, the levels of lysophospholipids, phosphatidylcholines, phosphatidylethanolamines, and ceramides, as well as other closely related lipid substances including fatty acids, diacylglycerols, and triacylglycerols, were observed to be significantly altered in AD animals (Zhang et al., 2020) and in plasma samples from preclinical AD and mild cognitive impairment AD (Peña-Bautista et al., 2022). Therefore, imbalances in the levels of phosphatidylcholines, fatty acids, and glycerides at different ages appear to be related to overactivation of phospholipases and diacylglycerol lipases, decreased anabolism of lysophospholipids in plasma and phosphatidylethanolamines in plasma and brain, and decreased anabolism of lysophospholipids in brain. The study suggests the possibility of creating lipid biomarkers for the early-stage AD diagnosis (Zhang et al., 2020). Further, in a recent investigation, the authors have performed an integrative multiscale network analysis, identify the modules, and then investigate the involvement of blood lipids and proteins in AD at a systems level and integrated these networks with established AD risk loci. Their result manifested that the ApoE 4 genotype and the five protein modules that positively regulate cytokine production, neutrophil-mediated immunity, and humoral immune responses were associated with an increased risk of developing AD. Also, the immune response and lipid metabolism-related AD risk loci were linked with the lipid modules of phospholipids, triglycerides, sphingolipids, and cholesterol esters (Xu J et al., 2020).

**FIGURE 3:**
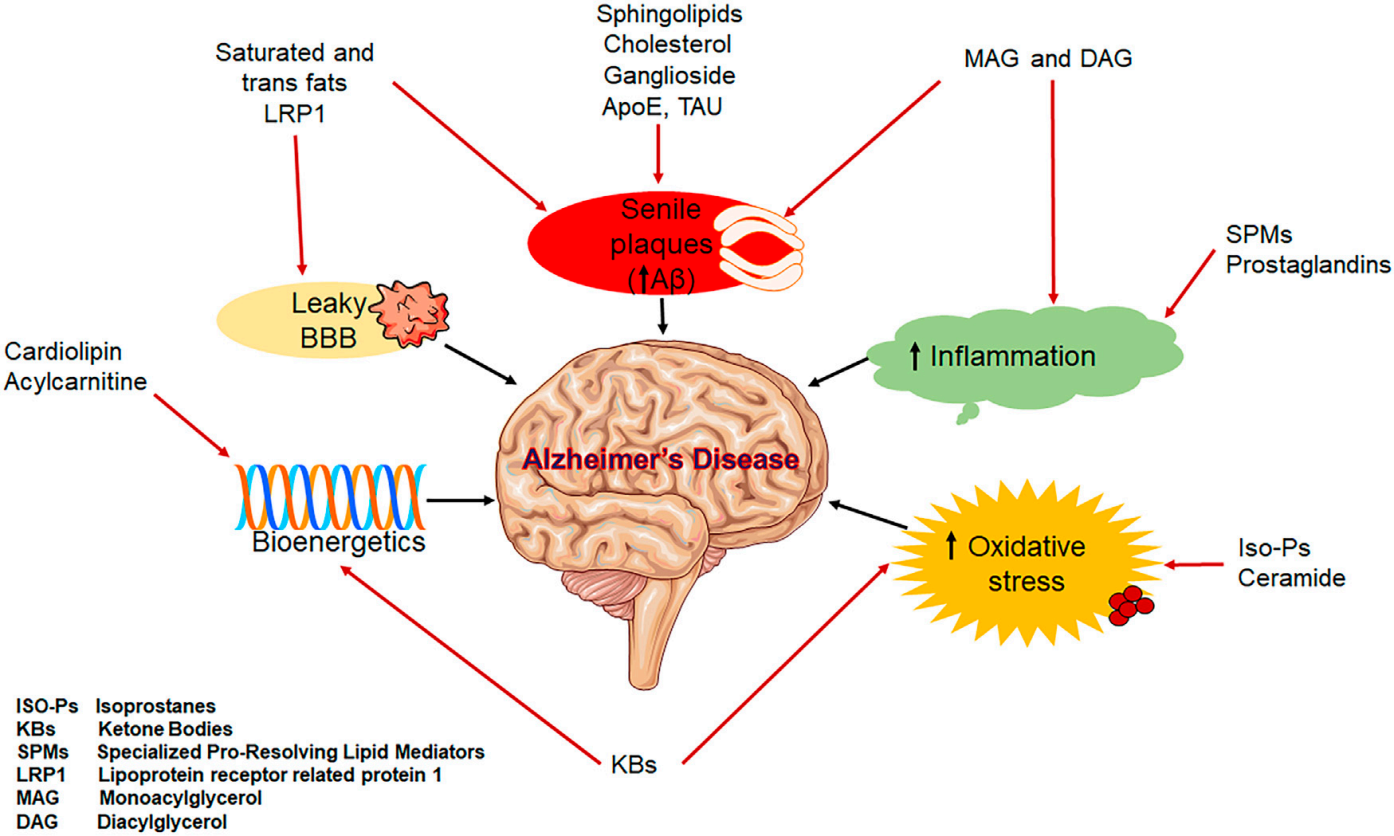
Schematic representation of lipids involved in AD.

Aging and the aetiology of AD are both strongly correlated with dysregulated lipid homeostasis (Figure 3). Alterations in the gut-brain axis, the neuronal signalling pathway, BBB disruption, mitochondrial dysfunction, oxidative stress, and inflammation are among the factors that link lipid dysregulation to AD. These factors collectively cause synaptic loss, which in turn impairs memory (Petot and Friedland 2004; Walter and van Echten-Deckert 2013). Some lipids have been postulated as biomarkers and play crucial roles in the pathophysiology of AD (Figure 3) (Kao et al., 2020).

It is now clear that genetic and environmental factors, including the presence of apolipoprotein and lipid transporter carrying status, affect cerebral lipid peroxidation and the composition of lipids in the brain, which are important predictors of AD pathogenesis (Bradley-Whitman and Lovell 2015). In fact, multiple research have used transgenic animals and cell culture to investigate the probable pathways through which ApoE4 is linked in the aetiology of AD (Huynh et al., 2017). These research include ones that investigated modifications in lipid metabolism which prevent neurite extension. Additionally, the lipid content of membrane lipid rafts is directly linked to amyloidogenesis, a significant pathogenetic component for AD (Ehehalt et al., 2003), which have a higher concentration of saturated fatty acids (FAs) than polyunsaturated fats (PUFAs) and sphingolipids, which operate as platforms for Aβ associations with tau and ApoE to facilitate the formation of A oligomers and hyperphosphorylation (Di Paolo and Kim 2011; Huynh et al., 2017). In addition, cortical and free unsaturated FAs induce the assembly of amyloid and tau filaments *in vitro* (Chiurchiù et al., 2022).

### Lipids involved in Alzheimer’s disease

A protein called ApoE, which is encoded on chromosome 19q 13.2, is involved in the transport of lipids, particularly cholesterol. Through the use of lipoprotein particles, ApoE facilitates the transfer of phospholipid and extracellular cholesterol. The human ApoE protein is comprised of 299 amino acids, and exists as three major isoforms namely: ApoE ε2, ε3 and ε4 (Lim et al., 2014). Cholesterol is crucial for cell membrane structure and function, especially in ion pumps and lipid rafts, which are specialised membrane microdomains that separate out specific cellular functions. Lipid rafts in particular offer the structural framework for signalling molecules and other proteins on the cell surface due to their increased cholesterol and saturated fat content compared to the nearby more fluid lipid bilayer membrane areas (Li et al., 2005; Vaya and Schipper 2007; Shepardson et al., 2011). In these cholesterol-rich lipid rafts, APP is cleaved by β-secretase as the final step in the synthesis of Aβ peptide. Because they are hydrophobic, peptides are likely to be affected by the lipid composition of membranes in both the synthesis and release processes. Membrane lipid composition contributes significantly to the pathophysiology of the disease for a variety of reasons, including this one. Numerous studies have demonstrated that variations in the amount of cholesterol in the lipid bilayer affect the way the APP is processed, which in turn affects how much Aβ is produced. More recently, it has been demonstrated that local increases in membrane cholesterol increase the cleavage of APP by the enzyme β-secretase (BACE1) and consequently increase the production of the amino acid. This is because the enzyme (β-secretase) and substrate are more closely localised in lipid rafts as a necessary consequence (Lim et al., 2014).

Ceramides, the key players in the metabolism of sphingolipids and lipid second messengers, have been linked to the pathophysiology and progression of AD *via* the production of Aβ. Through the stability of β-secretase, a crucial enzyme in the amyloidogenic processing of the APP, higher levels of ceramides directly increase Aβ. By activating the sphingomyelinases that catalyse the catabolic degradation of sphingomyelin to ceramide, the produced oligomeric and fibrillar Aβ stimulates a further increase in ceramide levels as part of a positive feedback loop (Jazvinšćak Jembrek et al., 2015). It has been hypothesised that ceramide plays a role in the neuronal cell death that causes AD. In a study, the authors have identified the altered (upregulated) levels of Cer16, Cer18, Cer20, and Cer24 in brains from patients suffering from AD. Further, the researchers have also identified the change in expression of some important genes involved in ceramide metabolism and found the elevated content of ASMase, NSMase 2, and GALC genes in samples obtained from patients with neuropathologic abnormalities (Filippov et al., 2012).

Endosomes are important cellular sorting compartments. They are membrane-bound vesicles that are typically differentiated into early, late, or recycling endosomes. They can transport proteins from the plasma membrane to the lysosome, or internally from the Golgi to the lysosome. Numerous lipid and protein studies have revealed endosomal dysfunction in AD as well as metabolic disorders (Di Paolo and Kim 2011). In addition, endosomal abnormalities have been discovered in AD, and crucially, these can be observed before amyloid and tau pathology in the neocortex. The endosomal-lysosomal pathway is involved in the proteolytic conversion of APP to Aβ. Drug approaches that target endosomes and the transport of Aβ are therefore receiving much attention of researchers (van der Kant et al., 2020).

### Lipidomics applications

Lipidomics is a specialised area of analytical chemistry and bioinformatics that uses mass spectrometric techniques to reveal the structures, roles, and dynamics of lipids in biological systems. Because of the wide variety of their structural and physiochemical characteristics, lipids, which have a central role in energy storage, signalling, and biofilm formations, play significant roles in a number of cellular activities. Lipidomics is the comprehensive study of biogenic lipid pathways, the large-scale profiling and quantification of biogenic lipid molecules, and the interpretation of their physiological importance based on analytical chemistry and statistical analysis. Lipidomics offers a method for finding significant biomarkers for the detection or treatment of human disorders in addition to offering insight into the physiological roles of lipid molecules. The most popular and efficient analytical methods for measuring and characterising lipids at the moment are those based on mass spectrometry. The field of mass-spectrometry-based lipidomics has been discussed with advantages and disadvantages in Table 2.

**TABLE 2:** Mass spectrometry-based techniques for lipidomics.

S.No.	Technique	Basis of application	Advantage	Disadvantage	References
1	LC based	*LC-MS or MS/MS (ESI, APCI, or APPI MS)*	Isomer and isobar separation, ion suppression reduction, sample matrix effects reduction, very sensitive targeted analysis; 2DLC-MS offline or online for thorough profiling analysis.	Low throughput, targeted analysis requiring previous knowledge (e.g., establishing SRM transitions), retention time drift, impacts of mobile phase (e.g., composition, salts, or additives), and extensive data processing are some of the problems that can arise.	(Hu and Zhang 2018)
		*MALDI MS*	Analysis of additional phospholipid classes is facilitated by the removal of strong PC peaks.	Low throughput and high ion source fragmentation.	(Köfeler et al., 2012; Xu T et al., 2020)
		*Ion mobility MS*	Separating and identifying isomeric and isobaric lipid species; To increase peak performance and signal-to-noise ratio (S/N), chemical noise that co-elutes on chromatography is resolved.; obtaining considerably cleaner fragment ion spectra by matching molecular ion and fragment ion spectra at a certain ion mobility drift period and chromatographic retention duration.	Loss of sensitivity; hard to compile with the time limit on the LC run.	Ivanova et al. (2009)
2	Shotgun based	*Tandem MS*	Straightforward; simple and fast; semiqualitative; quantitative.	Internal standard selection could be challenging, and the targeted MS/MS analysis might not be totally specific to the class of interest; Accurate quantification may be impacted by particular molecular species’ variable fragmentation kinetics; It may not be possible to discern structural identities and isobaric species.	(Hsu 2018)
		*High mass accuracy MS*	Analyses that are thorough and sensitive; accurate measurements of the weights of molecular ions and fragment ions that essentially rule out the possibility of false positive identification.	Differential responses of different species, particularly among non-polar lipid classes, need to be corrected for accurate quantitation; linear dynamic range of quantitation heavily depends on the instrument under experimental conditions. Differential fragmentation kinetics of various species may have an impact on quantification using multi-PIS or NLS data utilising the sum intensities of fragment ions.; unable to distinguish isomeric species having identical fragmentation patterns.	

The use of holistic metabolomic techniques is emerging to investigate the pathological characteristics underlying this neurodegenerative condition and to find potential diagnostic biomarkers due to the multifaceted nature of AD pathogenesis.

1) *LC-MS or MS/MS* (*ESI, APCI, or APPI MS*)*:* Normal-phase liquid chromatography, reversed-phase liquid chromatography, hydrophilic interaction liquid chromatography (HILIC), and mix-mode liquid chromatography all meet the various requirements of various lipid classes/species and have good reproducibility, high resolving power, and chromatographic enrichment.
2) *MALDI MS:* To remove the signal-suppressing lipid classes (such as PC) or overlapping species, separate the lipid classes beforehand using offline LC.
3) *Ion mobility MS:* The size, shape, charge, and mass separation of ionised molecules based on their various ion mobilities in low or high electric fields; a crucial adjunct to the LC separation of molecules and the MS separation of ions.

### Shotgun based lipidomics

Han and Gross were the ones who initially used the phrase “shotgun lipidomics”. Both subsequently used the method to look at the role of lipids in AD in the plasma and CSF. Shotgun lipidomics advances lipidomics by enabling both the high-throughput identification and quantification of lipids as well as the interaction between various lipid species and biological systems. Lipidomics is the study of cellular lipidomes derived from biological sources. Shotgun lipidomic approaches are high-throughput, simple, and sensitive, enabling large-scale analysis of very complex materials even though internal standards are needed for quantitation. Internal norms, nevertheless, are frequently elusive. When shotgun lipidomics is used with MALDI imaging, matrix and background interferences in the low mass range might make lipid identification difficult.

1) *Tandem MS:* Each lipid class of interest must have at least two additional internal standards added, and the tandem MS analysis must be exclusive to that lipid class.
2) *High mass accuracy MS:* The use of instruments with high mass resolution and mass accuracy to quickly and accurately perform production scans step by step within a whole mass region of interest; the extraction of multiple PIS or NLS from the acquired data array of the production spectra to identify individual species and quantify identified species through the sum of a molecular ion’s intensities in comparison to that of internal standards; or data-dependent acquisition to acquire product ion spectra for identification and well-resolved high mass accuracy full mass spectra for quantification.

### Studies related to metabolomics and proteomics

*Metabolomics:* Precision medicine is an emerging field of research that has great potential. Comprehensive study of metabolites in a biological specimen is what is meant by the term. It is the most recent systems biology technique where levels of small molecule metabolites in biological samples are measured using a variety of platforms. One of the most often used omic methods in clinical studies is proteomics, followed by metabolomics, epigenomics, and epigenetics. A significant amount of data from AD patients and healthy people of the same age is being produced through the development of omic platforms and advancements in bioinformatics. Therefore, in the coming years, omic studies will enable a significant advance in our understanding of AD on a number of fronts, including: I the identification of biomarkers to be used in the diagnosis or prognosis of the disease; ii) the advancement of our understanding of potential physio-pathological mechanisms; and iii) the development of novel and efficient therapeutic approaches. One of the most often used omic methods in clinical studies is proteomics, followed by metabolomics, epigenomics, and epigenetics. Individuals have distinctive metabolic signatures, and changes in metabolite levels can provide information about the disease state and the disorder’s underlying causes (Peña-Bautista et al., 2019).

The fact that cerebral hypometabolism develops in AD patients 20 years or more before clinical symptoms appear suggests that metabolic dysfunction plays a role in the development of AD. Although it only makes up 2% of total body weight, the brain is particularly dependent on glucose, using up 20% of all glucose-derived energy. In order to maintain energy homeostasis when glycolytic activities in the brain are compromised, compensatory systems switch to alternative fuel sources. Both CSF and plasma from AD patients had metabolic abnormalities, including neurotransmission and inflammation, although the energy pathways still saw the most significant changes. Similar results were also seen in numerous mouse models of AD, where female mice showed larger alterations in metabolic pathways associated to energy stress than male mice. By detecting long-term changes in the metabolic networks of CN, MCI, and AD patients, it is possible to develop panels of metabolic biomarkers and gain significant mechanistic insight into disease mechanisms (Wilkins and Trushina 2018).

## Conclusion

The recent failure of clinical trials aimed to control Aβ production has redirected the focus of preclinical drug discovery and academic research to the identification of new therapeutic targets and early causes of AD, including altered brain energetics and mitochondrial dysfunction. The use of metabolomics in AD research is gaining momentum because it enables the monitoring of changes in numerous interconnected networks that are crucial for comprehending complex metabolic abnormalities.
